# Quantitative Diagnosis Progress of Ultrasound Imaging Technology in Thyroid Diffuse Diseases

**DOI:** 10.3390/diagnostics13040700

**Published:** 2023-02-13

**Authors:** Jing Huang, Jiaqi Zhao

**Affiliations:** 1 Department of Ultrasound, Changzheng Hospital, Naval Medical University (Second Military Medical University), Shanghai 200003, China; 2 Department of Ultrasound, Shanghai Fourth People’s Hospital, School of Medicine, Tongji University, Shanghai 200434, China

**Keywords:** thyroid, diffuse disease, quantitative diagnosis, ultrasonography, intelligent imaging

## Abstract

High-frequency ultrasound (HFUS), the imaging modality of choice for thyroid screening, is most commonly used in the study of diffuse thyroid disease (DTD) with Hashimoto’s thyroiditis (HT) and Graves’ disease (GD). DTD can involve thyroid function and severely affect life quality, so early diagnosis is important for the development of timely clinical intervention strategies. Previously, the diagnosis of DTD relied on qualitative ultrasound imaging and related laboratory tests. In recent years, with the development of multimodal imaging and intelligent medicine, ultrasound and other diagnostic imaging techniques have gradually become more widely used for quantitative assessment of the structure and function of DTD. In this paper, we review the current status and progress of quantitative diagnostic ultrasound imaging techniques for DTD.

## 1. Introduction

Diffuse thyroid diseases (DTD) are relatively common in clinical practice. They are endocrine disorders in which the parenchymal structure of the thyroid gland is diffusely altered by a variety of etiologies, resulting in hyper- or hypothyroidism due to excessive synthesis and hypersecretion of thyroid-related hormones [1]. Among them, chronic lymphocytic thyroiditis (Hashimoto’s thyroiditis, HT) and toxic diffuse goiter (Graves’ disease, GD) are the two most common types that both fall into the category of autoimmune thyroid disease (AITD), account for 30% of all autoimmune diseases, and are T cell-mediated organ-specific autoimmune disorders [2]. These two are relatively similar in terms of immune-mediated disease mechanisms but differ in the degree of inflammation and the destruction of the thyroid gland, with GD having a milder inflammation of the thyroid gland, dominated by the serum antithyroid antibody (ATA)-induced hyperthyroidism with an overall diffuse enlargement of the thyroid gland complicated by hypermetabolism [3], whereas HT is dominated by various antibodies, which are produced by thyroid inflammation and the loss of immunological tolerance [4]. In some cases, hyperthyroidism and hypothyroidism may alternate [5]. Therefore, it is imperative and necessary to accurately diagnose, evaluate, and scientifically manage DTD as well as to provide precise treatment for different diseases.

The presence of DTD is usually accompanied by changes in thyroid function. Usually, hyperthyroidism diagnosis is straightforward and typically includes the combination of laboratory evaluation, physical findings, and historical symptoms [6], with raised serum thyroid hormones and suppressed serum thyroid-stimulating hormone (TSH) in almost all cases. Thyrotoxicosis can be easily diagnosed by high serum levels of thyroxine (T4) and triiodothyronine (T3) and a low serum level of TSH. Hyperthyroidism is confirmed by a high isotope (I-131 or Tc-99m) uptake by the thyroid gland, while in thyroiditis it will be low. The opposite is true for hypothyroidism. Thyroid function evaluation guidelines recommend measuring TSH first [7]. Also in those patients, numerous studies reemphasized the need for ultrasound as an essential part of the evaluation, as thyroid gland morphology could be variable.

High-frequency ultrasonography (HFUS) in conventional two-dimensional grayscale mode can provide diagnostic information about the morphology, structure, and internal echogenic changes of the thyroid parenchyma in a non-invasive and rapid manner, which is of great significance in guiding clinical intervention strategies. Based on this, combined with color Doppler flow imaging (CDFI), the distribution of blood flow in the thyroid parenchyma can be visualized dynamically, which can help improve the accuracy of qualitative diagnosis [8,9,10]. However, the conventional ultrasound imaging techniques mentioned above are mainly used for the preliminary determination of morphological, structural, and blood flow changes due to DTD. Such techniques can be unilateral in terms of qualitative diagnosis and are inevitably limited by the disparities between diagnosticians [11]. Therefore, in clinical practice, it is still necessary to closely integrate the patient’s clinical manifestations and laboratory test results to make a comprehensive judgment. With the intelligent development of multimodal medical imaging technology, new diagnostic ultrasound imaging techniques are gradually being applied to quantitatively assess the structure and function of DTD, which can guide individualized clinical treatment. In this paper, we review the current status and progress of quantitative diagnostic ultrasound imaging techniques for DTD.

## 2. Applications of Routine Technology for Quantitative Diagnosis by High-Frequency Ultrasound (HFUS)

### 2.1. Two-Dimensional Grayscale Ultrasound (2D GSUS)

The main objective is to observe the morphology and structure of the thyroid gland. As shown on 2D GSUS images, the normal thyroid parenchyma is surrounded by two layers of continuous and neat hyperechoic tegument, and the interior is isoechoic, dense, and even. Measurement of the thyroid lobe involves three measurements: the width, depth, and length; the volume is then calculated by the formula V = W × D ×L × π/6 (V—volume, W—width, D—depth, L—length). The total volume results from the sum of the two volumes (the isthmus is omitted unless its thickness is over 3 mm). According to the literature, the limits of normal thyroid volume are 10–15 mL for females and 12–18 mL for males. By quantifying the size and volume of the thyroid gland through 2D GSUS, the enlarged changes in the thyroid gland due to GD and its response to suppressive treatment can be clarified, and the radioiodine content can be strictly calculated based on the variation of size and volume [12].

DTD is commonly seen as diffuse abnormally reduced or increased echogenicity with coarse echogenic texture on 2D GSUS images, even with micro-nodule formation [13]. The GD is usually a diffuse enlargement of the parenchyma that is not homogeneous, and the echogenicity of thyroid is often decreased due to the hyperplasia, microfollicular pattern, increased vascularity, and reduced colloidal content [14]. The degree of hypoechoicity varies depending on the degree of pathologic changes and can be homogeneous/limited irregular patchy/diffuse and finely hypoechoic. The sonographic appearance of HT is characterized by decreased echogenicity, heterogeneity, hypervascularity, and the presence of hypoechoic micronodules with echogenic rims [15], which varies along with the degree of glandular involvement; antibody- or immune cell-mediated cytotoxic destruction is the underlying cause of morphologic and microscopic changes in the thyroid gland. In most patients with HT, the pathological basis of diffuse hypoechoic changes in the thyroid gland is the scattered or focal infiltration of a large number of lymphocyte-dominated inflammatory cells in the thyroid tissue and the formation of lymphocyte aggregates and germinal centers of variable sizes, small thyroid follicles with sparse colloid, oxyphilic changes of epithelial cells, and variable fibrosis [16]. Mazziotti and colleagues analyzed 89 patients with Hashimoto’s thyroiditis and 40 healthy controls, and they found that thyroid echogenicity evaluated by grayscale (GWE) quantitative analysis was lower in patients with the disease when compared to the normal thyroid (61.9 ± 8.3 GWE vs. 71.9 ± 3.1 GWE; *p* = 0.01) [17]. In some patients with HT, multiple scattered hypoechoic micronodules can be seen, ranging in size from 1–7 mm and surrounded by fibrotic or calcified hyperechoic margins, and semi-quantitative interpretation of HT by image features is highly specific, with a positive predictive value of 95% [18].

### 2.2. Doppler Ultrasound

Based on the full analysis of 2D GSUS images, combined with the distribution of blood flow in the parenchyma of the thyroid gland shown on color Doppler flow imaging (CDFI), the abundance of blood supply in the parenchyma of the thyroid gland can be visually evaluated. The peak systolic velocity (PSV), end-diastolic velocity (EDV), resistance index (RI), etc., can be measured by spectral pulse wave Doppler (PW) ultrasound and compared with normal thyroid blood flow. By combining three-dimensional quantitative energy Doppler parameters such as vascularization index (VI), flow index (FI), and vascularization flow index (VFI), the changes in hemodynamic parameters in DTD can be assessed quantitatively. However, there is no consensus about the effectiveness of blood flow in diagnosing thyroid diseases. Sometimes the blood flow does not reflect the actual condition of the thyroid disease, such as the thyroid blood flow of some DTDs displaying an uncharacteristic distribution that is nevertheless obviously different from the blood flow distribution around lesions of adenoma and nodular goiter [19]. Observing the blood flow of the focused-upon area can better reflect the disease process and is a good index to guide the treatment.

Taking GD and HT as examples, the visual flow grading method [20] is commonly used for Adler’s semi-quantitative evaluation [21] of the blood flow distribution shown on CDFI of these two common DTDs. The grading system can be explained as follows: Grade 0: no blood flow signal in the normal thyroid parenchyma, with only the larger vascular branches showing colored blood flow; Grade I: dotted, striped, and small patchy colored signals scattered in the thyroid parenchyma, mostly without fusion, with the colored area being <1/3 the size of the thyroid area; Grade II: patchy blood flow signal scattered in the thyroid parenchyma, partially fused into a large colored mosaic, with the colored area taking up 1/3 to 1/2 of the thyroid area; and Grade III: the thyroid gland is filled with colored blood flow signals, with a large fused multicolored mosaic and colored area taking up >2/3 of the thyroid gland area, including the “thyroid inferno” [22], which is a diffuse distribution of pulsating, multicolored, flickering blood flow signals in the gland. In addition, because the inferior thyroid artery (ITA) is superficial, straight, easy to visualize, and supplies the lower one-third of the thyroid gland, the PSV and other parameters can be measured more easily, which can quantitatively reflect the hemodynamic status of the thyroid parenchyma to a certain extent. Previous studies have shown that combined diagnosis via CDFI and spectral Doppler ultrasound can distinguish GD from hyperthyroidism caused by other factors with a high specificity [23]. Banaka et al. [24] found that the PSV of the left inferior thyroid artery was the most accurate acoustic parameter for the diagnosis of autoimmune thyroid disease. In their study, a PSV greater than 61.65 cm/s in the left inferior thyroid artery had a sensitivity of 82.8% and a specificity of 86.9% for differentiating HT from GD. Malik et al. [25] found that patients with GD had significantly higher mean PSV-ITA values than those with thyroiditis. At a mean PSV-ITA cutoff value of 30 cm/s, PSV-ITA discriminated GD from thyroiditis with a sensitivity of 91% and a specificity of 89%.

According to the blood flow grading shown by CDFI in different conditions in patients with diffuse thyroid lesions, it can be used as a quantitative indicator to assist in evaluating the perfusion status of the thyroid gland, not only for quantitative diagnosis but also for quantitative assessment of treatment effects as well as recurrence prediction. The authors of [26] show that by evaluating the PSV of the ITA, Doppler ultrasound was useful for monitoring the therapeutic response of GD patients after treatment with radioiodine. They found that the mean PSV before I131 was 90.06 ± 44.13 cm/s and decreased significantly over time (*p* < 0.001). Six months after the therapeutic dose, the mean PSV was 32.95 ± 16.36 cm/s. Another observational study [27] of 22 women with HT who were planning and later achieved pregnancy or were confirmed as pregnant showed that TSH (β = 0.507, *p* = 0.008) and ITA-PSV (β = −0.362, *p* = 0.047) were independently associated with increased LT4 dosage. In predicting disease recurrence, Saleh et al. [28] found that by measuring ITA-PSV and volumetric flow rate values (VFR, calculated by multiplying the cross-sectional area of the thyroid vessel with time-averaged mean blood velocity), mean ITA-PSV and VFR values, as well as thyroid volume measured at the time of diagnosis, were significantly higher (139 cm/s, SD 46; 195 mL/min, SD 170; 52 mL, SD 18) in patients who relapsed after drug treatment compared with patients in remission (71 cm/s, SD 27; 67 mL/min, SD 61; 25 mL, SD 13). The recurrence of disease could be predicted with a sensitivity of 71% and a specificity of 100% based on thyroid blood flow measurements. All of these quantitative ultrasound techniques facilitate better treatment selection and adjustment of treatment cycles by clinicians, leading to higher long-term remission rates with antithyroid medications.

## 3. Application of New Technology for Quantitative Diagnosis by High-Frequency Ultrasound

### 3.1. Microvascular Ultrasound Imaging

In some cases, the sensitivity of CDFI in detecting low-velocity microvascular flow may be limited due to the significant increase of microvascular flow in the overall parenchyma of the thyroid gland in patients with GD. Superb microvascular imaging (SMI), an emerging ultrasound Doppler technique with high resolution and a high frame rate, is able to show microperfusion changes in the lesion area with a duct diameter larger than 0.1 mm in high definition [29,30]. With the help of SMI, Abidin et al. [31] found that the median level of vascularization index of 12 (2.3–32.1) in the thyroid parenchyma of GD patients was significantly higher than that of 5.04 (1.1–10.8) among an asymptomatic group, *p* < 0.001. When the median vascularization index threshold was set at 6.3, the sensitivity and specificity of GD diagnosis were 83.8% and 70%, respectively. Another study [32] about the evaluation of pediatric GD and HT showed that by evaluating vascularity indices (VIs), patients with GD were found to have a significantly higher median VI (right, 25%; left, 26%) compared to patients with HT (right, 11%; left, 13%) and control participants (right, 8%; left, 8%). The optimal cutoff VI value for distinguishing between HT and GD was 17.35%, with a sensitivity, specificity, and diagnostic accuracy of 85.3%, 78.4%, and 81.7%, respectively. Therefore, the vascularization index obtained by superb microvascular imaging can be used as a quantitative index for measuring the diagnosis of parenchymal vascularization in GD.

Another newly introduced high-resolution energy Doppler modality—advanced dynamic flow (ADF)—is used to quantitatively detect thyroid blood flow (TBF), and the microvascular flow information of the thyroid parenchyma can also be quantified after the advanced dynamic flow/region of interest (ADF/ROI) ratio is calculated by special software. It was shown that TBF values in patients with GD were consistently >4%, significantly higher than the figures among counterparts with painless thyroiditis, subacute thyroiditis, or normal controls (*p* < 0.0001), and that 4% could be used as the dividing line to distinguish destructive thyrotoxicosis from GD [33]. As a result, these new techniques employed in blood flow imaging will facilitate the further assessment of parenchymal blood flow in the thyroid gland and provide an accurate understanding of the extent of DTD lesions for the selection of optimal treatments.

### 3.2. Three-Dimensional Ultrasound (3D US)

Three-dimensional images can display the spatial morphological structure of thyroid lesions, which is conducive to the intuitive understanding of the image by laymen. Due to the development of multimodal ultrasound technology, new composite ultrasound technologies, such as three-dimensional color Doppler and three-dimensional ultra-microvascular imaging, are becoming increasingly better established. Three-dimensional color Doppler imaging demonstrates the spatial anatomical patterns and relationships of the vascular architecture of the thyroid lesion, which gives further diagnostic information with the accurate presentation of both the number and distribution density of vessels within the lesion [34]. Karakas et al. [35] measured several parameters related to thyroid blood flow by the superior thyroid artery (STA) and common carotid artery (CCA) combined with triplex doppler ultrasonography (TDU). The analysis revealed that the STA-PSV, STA-EDV, peak systolic velocity ratio (PSVR), and end-diastolic velocity ratio (EDVR) values were significantly higher in patients with thyrotoxicosis (including GD and HT) compared to the normal group. Chiou et al. [36] evaluated the intra-observer and inter-observer reproducibility of three-dimensional (3D) power Doppler ultrasonography with the virtual organ computer-aided analysis (VOCAL) program, measuring thyroid volume and vascular indices (including the vascularization index, flow index, and vascularization–flow index) in patients with diffuse thyroid disorders. They found that the thyroid volume and three vascular indices showed excellent reproducibility in the AITD group (23 Graves’ disease, 21 Hashimoto’s thyroiditis), and that the vascularization index is the most reliable parameter of all. It has distinct advantages in the inclusive and accurate assessment of blood supply distribution within diffuse thyroid lesions and plays a significant role in the practices of quantitative diagnosis.

### 3.3. Ultrasound Elastography (USE)

This new technique, which was first proposed in 1991 by Ophir et al., enables non-invasive assessment of the elastic texture of parenchymal tissue [37] and was first used in the thyroid in 2005 by Lyshchik et al. [38]. This technique reflects the stiffness of the lesioned tissue by measuring its elasticity coefficient and it can be used to assess changes in the parenchymal elastic mechanical characteristics due to DTD [39]. Current USE techniques utilize different excitation methods: manual compression (by hand or using cardiovascular pulsation or respiratory motion), acoustic radiation force impulse (ARFI), or external mechanical vibration [40]. Strain imaging and shear wave imaging are classified according to the physical quantities detected, which include the elastic strain rate ratio (%), Young’s modulus (Kpa), shear wave velocity (m/s), etc.

Comparing the strain ratio of thyroid nodules to sternocleidomastoid muscle in the same strain elastic ultrasound images and calculating the elastic strain ratio (SR) values in ascending order yields the following hierarchy: control group ≤ hyperthyroidism group < Hashimoto’s thyroiditis group. Yang et al. [41] found that the difference between the groups bears great statistical significance. In addition, Cepeha et al. [42] found that a mean SR value above 1.64 predicted the presence of chronic autoimmune thyroiditis with a sensitivity (Sen) of 69%, specificity of 92%, positive predictive value (PPV) of 95.4%, negative predictive value (NPV) of 54%, and area of receiver operating characteristic (AUROC) of 0.87. It has been proven that the quantitative assessment of thyroid lesions by SR is genuinely feasible.

With virtual touch tissue imaging (VTI) of ARFI technology and virtual touch tissue quantification (VTQ), Sporea et al. [43] analyzed the elastic distribution characteristics of thyroid tissue in healthy subjects, GD patients, and chronic autoimmune thyroiditis (CAT) patients. The VTQ technique was found to be able to quantitatively diagnose and quantitatively assess the disease process in normal thyroid and HT, GD, and subacute thyroiditis in DTD. The results showed that the thyroid stiffness (TS) values assessed by ARFI were significantly lower in the healthy population (2.00 ± 0.40 m/s) than in patients with GD (2.67 ± 0.53 m/s), *p* = 0.0001, and lower than in patients with CAT (2.43 ± 0.58 m/s) *p* = 0.0002; predictions were obtained for DTD with an optimal TS threshold of about 2.36 m/s (sensitivity 62.5%, specificity 79.5%, positive predictive value 87.6%, negative predictive value 55.5%), with an accuracy of 72.7%.

It has been reported in [44] that shear wave elastography (SWE) was used to discriminate between normal thyroid condition and DTD with a positive diagnostic predictive value of >90% when the measured shear wave velocity threshold was >2.53 m/s. Shear wave velocity values significantly differed between the two groups of patients with different types of DTD (GD and HT) (2.07 ± 0.44 m/s vs. 2.68 ± 0.50 m/s). Moreover, SWE could be considered very helpful in accurately predicting the presence of DTD (AUC approximately 0.804). In addition, Kim et al. [45] found that for DTD, the critical mean value of Young’s modulus was 27.6 kPa, and the maximum value was 41.3 kPa, measured using the carotid artery as the source of internal pressure. More importantly, there was higher specificity in using the mean elasticity value as a quantitative diagnostic criterion for DTD, compared with the application of the maximum elasticity value. Furthermore, in a study of children and adolescents with diffuse thyroid disorders, Hazem et al. [46] found mean Young’s modulus values of 10.9 ± 1.78 kPa, 15.31 ± 2.95 kPa, and 17.26 ± 4.2 kPa, respectively, in normal participants and patients with HT or GD. Statistically significant differences were found between the mean Young’s modulus values of normal subjects and those of patients with DTD, as well as between the mean values of patients with different types of DTD. The optimal Young’s modulus threshold to distinguish normal thyroid condition from DTD in adolescents was 12.8 kPa, whereas the critical value for distinguishing HT from GD was 17.8 kPa.

### 3.4. Contrast-Enhanced Ultrasound (CEUS)

Contrast-enhanced ultrasound (CEUS) is implemented to examine the microflow perfusion information in lesions following the peripheral intravenous injection of ultrasound contrast agent (UCA). It is considered to be the third revolution in ultrasound medical technology and was first applied to the liver and subsequently widely used in the examination of superficial organs such as the thyroid, breast, and lymph nodes [47]. However, CEUS is mostly used for screening and identifying nodules or tumors in thyroid lesions, but more needs to be done for DTD. Recent studies have reported on the diagnostic utility of CEUS for the benign and malignant diagnosis of concomitant thyroid nodules in the context of HT. For example, Zhao et al. [48] found that in HT patients, malignant and benign thyroid nodules differed considerably in peak intensity (*p* = 0.002) and enhancement pattern (*p* < 0.001). The sensitivity, specificity, positive predictive value (PPV), negative predictive value (NPV), and accuracy of heterogeneous enhancement were 97.6%, 85.7%, 93.0%, 94.7%, and 93.5%, respectively. Heterogeneous enhancement is effective in the diagnosis of malignant thyroid nodules coexisting with HT. Ma et al. [49] quantified the CEUS features of 85 histopathologically confirmed thyroid nodules using five parameters:—the rising time (RT), time to peak (TTP), area under the curve (AUC), maximum intensity (Imax), and mean transit time (mTT)—and discovered that the quantitative parameters of CEUS, especially the Imax and AUC parameters, are valuable in diagnosing benign and malignant thyroid nodules. Malignant thyroid nodules showed significantly lower Imax (Z = −7.08, *p* = 0.01) and AUC (Z = −2.03, *p* = 0.04) values than normal thyroid tissue, whereas benign nodules had considerably higher Imax (Z = −1.35, *p* = 0.02) and AUC (Z = −0.21, *p* = 0.02) values. With the widespread use of CEUS, the quantitative studies related to DTD are expected to be further expanded.

## 4. Applications of Other Quantitative Diagnostic Imaging Technology

### 4.1. Computed Tomography (CT) and Magnetic Resonance Imaging (MRI)

Some scholars have suggested certain limitations of the ultrasound examination of the thyroid gland [50], such as high diagnostic subjectivity and low efficacy, whereas high-resolution rapid computed tomography (CT) and magnetic resonance imaging (MRI) have long since proven themselves sensitive and reliable in appropriate applications. CT and MRI not only provide essential information about the deep extension of clinically detected masses, they can also delineate additional clinically unsuspected lesions. The excellent tissue characterization of MRI scans can lead to an accurate diagnosis of many benign processes as well [51,52]. Therefore, specific CT or MRI features of DTD may aid in the diagnosis of related diseases. Hye Jin Baek et al. [53], through measurement, identified a great disparity in mean CT values (HU) of thyroid parenchyma on non-enhanced and contrast-enhanced CT images between control groups and patients with DTD. Using a cutoff value of <103 HU, the diagnostic accuracy of non-enhanced CT for DTD was 75.6%, with a specificity of 63.6%, a positive predictive value of 51.8%, and a negative predictive value of 83.5%. On contrast-enhanced CT images, a cutoff value of <205 HU resulted in a diagnostic accuracy of 67.9%, with a specificity of 56.3%, a positive predictive value of 44.5%, and a negative predictive value of 77.3%.

Mikio Tezuka et al. [54] demonstrated that although no prominent difference was found between T1- and T2-weighted thyroid images, the apparent diffusion coefficient (ADC) values were significantly higher in patients with GD than in patients with subacute thyroiditis and HT. Using a diagnostic threshold of 1.82 × 10^−3^ mm^2^/s, the quantitative diagnosis of GD had a sensitivity of 75% and specificity of 80%. Thus, quantitative diagnosis of thyroid CT values and MRI ADC values can assist in differentiating diffusely diseased thyroid tissues in some ways.

However, the application of MRI in the quantitative diagnosis of DTD is rather limited by the convenience, affordability, and reproducibility of MRI equipment. Indeed, CT and MRI are both rarely used in the diagnosis of DTD in daily clinical practice. Obviously, they have a unique advantage in complex anatomical structures and areas, such as the orbit, base of the skull, deep space in the neck, etc., such that using them just for DTD diagnostics seems somewhat excessive.

### 4.2. Thyroid Scintigraphy

Now, it is well known that thyroid gland iodine uptake is attributed to the sodium–iodide symporter (NIS) [55]. Normal thyroid tissue is characterized by the unique capability of its follicular cells to trap and to process iodine (I^2^), which is subsequently incorporated into thyroglobulin (Tg)-bound thyroid hormones. Because iodine plays a major role in the physiology and pathophysiology of the thyroid gland, iodine and iodine analogues (i.e., NIS-targeting radiopharmaceuticals) are well suited for thyroid imaging and radioiodine uptake (RAIU) study [56]. Concerning thyroid function evaluation, thyroid cell iodine uptake remains widely used by means of thyroid scintigraphy and radioiodine uptake testing. The current role of nuclear scintigraphy using Tc-99m pertechnetate or I-123/I-131 is adjunctive rather than as a first-line diagnostic method, and there are not enough studies to justify its routine use [57]. However, it not only gives valuable information regarding thyroid anatomy, but also it gives excellent functional information about the gland. There are specific scintigraphy patterns for GD and other DTDs. The radioiodine uptake test (RAIU) quantifies the global iodine metabolism within the thyroid gland as reflected by the radiopharmaceutical accumulation. Therefore, thyroid scintigraphy with either Tc-99m pertechnetate scintigraphy or radioiodine is useful to characterize different forms of hyperthyroidism and provides information for planning radioiodine therapy [58].

Both thyroid scintigraphy and RAIU testing are used to differentiate between productive thyrotoxicosis (i.e., hyperthyroidism) and destructive thyrotoxicosis (i.e., acute and subacute thyroiditis) and factitious thyrotoxicosis. However, the RAIU test has a very limited role for the diagnosis of thyroid disorders [56]. It provides essential information in hyperthyroid patients scheduled for therapy with I-131 [59]. In contrast, thyroid scintigraphy has a wider range of applications. Research has shown that there is very good agreement between scintigraphy diagnosis and ultrasonography. In many cases, scintigraphy provides considerably more functional and anatomic details than ultrasound [60].

Tc-99m uptake was found to be 0.97–40.1% in GD, 0.15–0.8% in HT, and less than 0.5% in all patients with subacute thyroiditis [61]. Another study [62] showed that it was useful to discriminate between GD and painless thyroiditis, with a cutoff value of 1.0% resulting in a diagnostic sensitivity and specificity of 96.6% and 97.1%, respectively. Moreover, Tc-99m pertechnetate thyroid scintigraphy can be used to predict the clinical outcomes of radioiodine therapy for GD. A study showed that a Tc-99m pertechnetate uptake above 18.4% is a significant predictor of treatment failure, and these patients should receive a higher radioiodine dose in this scenario [63]. The rate of Tc-99m pertechnetate uptake helps quantify the severity of GD and its response to drug therapy and can be used to scientifically determine the timing of antithyroid drug treatment in clinical practice. There is growing evidence that quantitative diagnosis of imaging technology offers unique advantages in the field of diagnosis and treatment of DTD. The application of high-frequency ultrasound and other quantitative imaging diagnosis technologies in diffuse thyroid diseases are briefly shown in Table 1.

### 4.3. Positron-Emission Tomography/Computed Tomography (PET/CT)

Incidental uptake of 2-[18F]-fluoro-2-deoxy-D-glucose (F-18 FDG) in the thyroid gland is not uncommonly encountered in day-to-day practice of oncological 18F-FDG positron-emission tomography/computed tomography (PET/CT), but its significance can be a challenge to evaluate due to its different causes. A systematic review shows that diffuse thyroid uptake of PET tracers is a relatively frequent event, ranging from 0.4 to 46.2%, and it is commonly related to benign disease. Among all pathological causes of diffuse F-18 FDG uptake in the thyroid, benign diseases such as chronic thyroiditis, GD, and adenomatous goiter are the most common conditions [64]. Therefore, diffuse increased thyroid F-18 FDG uptake can be informative about the presence of DTDs. However, further investigation and clinical evaluation are required for the correct diagnosis. Just like CT and MRI, PET/CT is also limited by the convenience, affordability and reproducibility of the equipment for the diagnostic application of DTD, and is consequently used rather sparingly in practice.

## 5. Summary and Outlook

**Figure 1:**
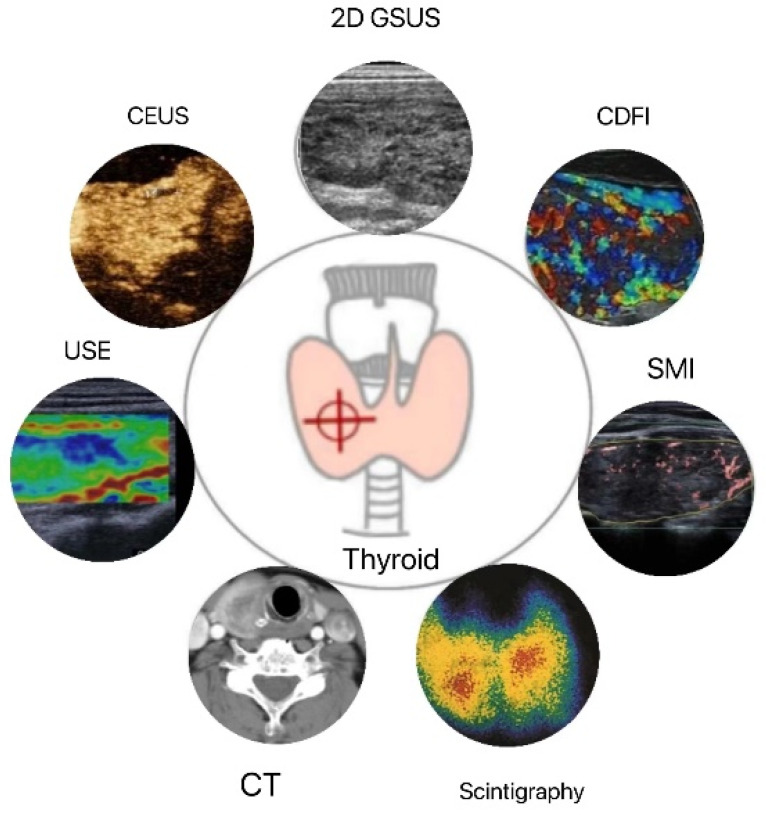
Applications of thyroid quantitative diagnostic imaging technology. 2D GSUS: two-dimensional grayscale ultrasound; CDFI: color Doppler flow imaging; SMI: superb microvascular imaging; CEUS: contrast-enhanced ultrasound; USE: ultrasound elastography; CT: computed tomography.

The combination of multimodal ultrasound imaging techniques can compensate for the deficiencies of a single diagnostic tool (Figure 1). With the continuous advancement of ultrasound technology, quantitative ultrasound is gradually gaining popularity for quantitative assessment of the structure and function of DTD, reducing the previous limitations of relying primarily on the subjective interpretation of images and qualitative diagnosis by an ultrasonographer. In recent years, the development of artificial intelligence (AI) has contributed to tremendous progress in the fields of intelligent recognition and quantitative diagnosis of ultrasound images. Recently, computer-aided diagnosis (CAD) technology [65,66] has been utilized to extract and analyze texture features from 2D grayscale ultrasound images of DTD, thereby obtaining more detailed features of ultrasound images for patients with DTD that cannot be distinguished by the human eye, which is of great clinical utility.

In the future, more inclusive analysis and quantitative diagnosis of DTD are likely to benefit from collecting and matching multimodal and multicenter thyroid ultrasound datasets with clinical, test, pathological, and other imaging data. What is more important is that the construction of a lesion diagnosis prediction model can offer guidance to the clinical formulation of custom-tailored individual treatment plans, which indicates a broader application for prognosis and efficacy assessment.

## Figures and Tables

**Table 1:** A brief summary of the application of high-frequency ultrasound (HFUS) and other quantitative imaging diagnosis technologies in diffuse thyroid diseases.

**Technology**	**Quantitative Methods**	**Value**	**Ref.**
2D GSUS	Volume	10–15 mL, female, normal	[12]
		12–18 mL, male, normal	
	Grayscales (GWE)	71.9 ± 3.1 GWE, HT *	[17]
		61.9 ± 8.3 GWE, normal	
CDFI	Adler’s semi-quantitative evaluation	Grade I–IV	[21]
	PSV-ITA	61.65 cm/s, HT * & GD *	[24,25]
		30 cm/s, GD * & thyroiditis	
		90.06 ± 44.13 cm/s, before I131	[26]
		32.95 ± 16.36 cm/s, 6 months later	
	PSV-ITA & VFR	139 cm/s, 195 mL/min, relapse patients	[28]
		71 cm/s, 67 mL/min, remission patients	
SMI	Vascularization index	12 (2.3–32.1), GD *	[31,32]
		5.04 (1.1–10.8), normal	
		17.35, pediatric GD * & HT *	
ADF	ADF-TBF	>4%, GD * & thyroiditis	[33]
3D US	STA/CCA-TDU	STA-PSV, STA-EDV, PSVR, EDVR	[35,36]
	3D US and VOCAL	Vascularization index	
USE	Strain elastic ultrasound	Strain ratio (SR)	[41,42]
		1.64, CAT *	
	ARFI-VTI/VTQ	TS 2.36 m/s, DTD *	[43]
	SWE	Shear wave velocity 2.53 m/s	[44,45]
		Young’s modulus 27.6 kPa	
CEUS	CEUS	Identification of nodules or tumors	[46]
CT	Mean CT values	103 HU, non-enhanced CT *	[53]
		205 HU, contrast-enhanced CT *	
MRI	ADC values	1.82 × 10^–3^ mm^2^/s, GD *	[54]
Thyroid Scintigraphy	Tc-99m uptake	0.97–40.1%, GD *	[61]
		0.15–0.8%, HT *	
		<0.5%, subacute thyroiditis	

* GD: Graves’ disease; HT: Hashimoto’s thyroiditis; DTD: diffuse thyroid disease; CAT: chronic autoimmune thyroiditis.

## Data Availability

Not applicable.
